# Evaluation of GPT-5 for Esophageal Cancer Staging Using Fluorodeoxyglucose Positron Emission Tomography Maximum-Intensity Projection Images: Comparative Pilot Study

**DOI:** 10.2196/86630

**Published:** 2026-02-23

**Authors:** Hiroki Maruyama, Yoshitaka Toyama, Yuya Araki, Kentaro Takanami, Masato Ito, Yumi Nakajima, Kei Takase, Takashi Kamei

**Affiliations:** 1 Department of Surgery Graduate School of Medicine Tohoku University Sendai Japan; 2 Department of Imaging and Anatomy for Groundbreaking Education Collaborative Research Graduate School of Medicine Tohoku University Sendai Japan; 3 Department of Diagnostic Radiology Tohoku University Hospital Sendai Japan; 4 School of Medicine Tohoku University Sendai Japan; 5 Department of Diagnostic Radiology Osaki Citizen Hospital Osaki Japan; 6 Department of Diagnostic Radiology Tohoku Medical and Pharmaceutical University Sendai Japan

**Keywords:** generative artificial intelligence, large language models, LLMs, 18F FDG-PET imaging, fluorodeoxyglucose positron emission tomography, esophageal cancer staging, radiology report automation

## Abstract

**Background:**

Accurate esophageal cancer staging relies on ^18^F fluorodeoxyglucose positron emission tomography (^18^F FDG-PET), but its interpretation is complex and time-intensive. This diagnostic burden is exacerbated by significant workforce shortages in both radiology and surgery, thus necessitating automated support systems. The emergence of advanced large language models (LLMs) has raised expectations for their potential to fulfill this role in complex medical tasks.

**Objective:**

We evaluated the diagnostic accuracy of LLMs for staging esophageal cancer using ^18^F FDG-PET images, with a focus on their ability to assess lymph nodes (LNs; clinical N [cN]) and distant metastases (clinical M [cM]) for automated radiology reporting.

**Methods:**

This retrospective study included 120 consecutive adult patients who were diagnosed with esophageal squamous cell carcinoma and underwent ^18^F FDG-PET/computed tomography at Tohoku University Hospital between January 2019 and December 2021. Patients with prior treatment, nonsquamous cell carcinoma histology, or blood glucose levels ≥200 mg/dL were excluded. Frontal maximum-intensity projection positron emission tomography images were extracted, standardized, and analyzed along with information regarding the tumor location. Six LLMs (GPT-5, GPT-4.5, GPT-4.1, OpenAI-o3, -o1, and GPT-4 Turbo) and 4 blinded human evaluators (a nuclear medicine specialist, a gastrointestinal surgeon, and 2 radiology residents) assessed the presence of thoracic and abdominal LN metastases on a region-level basis and determined cN and cM staging on a patient-level basis. The model analyses were performed using the application programming interface in a zero-shot setting. Radiology reports served as the reference standard. Diagnostic agreement and accuracy were evaluated using Cohen κ and the Cochran Q test. Additionally, to account for the class imbalance in the dataset, the Matthews Correlation Coefficient was calculated as a robust metric for binary classification performance. Post hoc McNemar tests were performed with Bonferroni correction; statistical significance for pairwise comparisons was set at *P*<.0083 (adjusted from *P*<.05) using JMP Pro (version 18.0; SAS Institute Inc).

**Results:**

The average accuracy was 41/120 (34%) to 94/120 (78%) for LLMs and 72/120 (60%) to 102/120 (85%) for physicians, with significantly higher accuracy for physicians (*P*<.05) in the thoracic LN, abdominal LN, and cN stages. Interrater reliability was slight to fair for LLMs (κ: –0.07 to 0.25) and fair to substantial for physicians (κ: 0.27 to 0.74). Matthews Correlation Coefficient scores were consistently higher for physicians (0.28 to 0.75) than for LLMs (–0.07 to 0.32). Among the LLMs, GPT-5 demonstrated the highest overall accuracy, with newer LLMs showing improved diagnostic accuracy when compared with previous models in identifying abdominal LN metastases and cM staging, though they showed weaker consistency for cN staging. For example, in thoracic LN detection, GPT-5 achieved 76/120 (63%) accuracy, whereas other LLMs achieved 72/120 (60%) or lower accuracy.

**Conclusions:**

Although current LLMs have not yet reached physician-level accuracy in comprehensive staging, recent models show promise in assisting with specific diagnostic tasks.

## Introduction

Esophageal cancer remains one of the most challenging malignancies to manage. According to the most recent GLOBOCAN statistics and Japanese cancer registry data, it poses a significant global health burden [1,2]. Management requires multidisciplinary expertise that spans complex surgical procedures, perioperative care, and advanced imaging interpretation. Esophagectomy is among the most invasive oncologic surgeries, and optimal patient outcomes depend on accurate staging, meticulous operative planning, and coordinated care.

Surgical services face a mounting workforce shortage: the Association of American Medical Colleges 2024 national projection estimates a shortfall of 10,100 to 19,900 surgeons by 2036 [3], and a nationwide Japanese survey reported that over half of teaching hospitals already experience surgeon shortages—even in densely populated prefectures [4]. In parallel, radiology faces both workforce shortages and escalating workload: the 2023 Workforce Census for the United Kingdom reports a 30% shortfall of clinical radiologists [5], while the volume of image data per study has surged markedly, compounding reporting demands [6,7].

Against this backdrop, fluorodeoxyglucose positron emission tomography/computed tomography (^18^F FDG-PET/CT)—a cornerstone of preoperative staging in esophageal cancer—is notably time-consuming and complex to interpret [8,9], requiring integration of functional and anatomical information. This adds to the workload of both surgeons, who must incorporate imaging findings into surgical planning, and radiologists, who must provide comprehensive and timely reports for multidisciplinary decision-making.

International guidelines, including the American College of Radiology Appropriateness Criteria and the European Society for Medical Oncology recommendations, endorse ^18^F FDG-PET/CT for baseline staging and selected follow-up in esophageal cancer [10,11]. ^18^F FDG-PET/CT is recognized not only for its diagnostic utility in detecting distant metastases and assessing nodal involvement but also for its significant prognostic value in oncology, as metabolic parameters often correlate with patient outcomes [12,13].

Generative artificial intelligence (AI), a subset of AI capable of creating new content, has revolutionized various fields. Within this domain, large language models (LLMs) are deep learning algorithms trained on massive datasets to understand and generate human-like text. Recently, the evolution of these models into multimodal large language models, which can process and interpret both text and images simultaneously, has expanded their potential applications in health care. While previous research has demonstrated the utility of AI in medical tasks such as summarizing radiology reports or passing medical licensing examinations [14], the application of general-purpose multimodal large language models to complex image interpretation remains limited. Most prior studies have focused on anatomical imaging modalities like plain radiography or CT for simple classification tasks [15-18]. There is a paucity of research evaluating whether these models can perform high-level clinical reasoning, specifically TNM staging based on functional nuclear medicine imaging (^18^F FDG-PET). Against this backdrop, and with the release of GPT-5, the latest publicly available LLM from OpenAI, we report the first evaluation of the medical image interpretation capabilities of this model. We compared its diagnostic accuracy in esophageal cancer staging with that of physicians and other state-of-the-art models.

## Methods

### Study Design

We adhered to the guidelines outlined in the checklist for AI in medical imaging to ensure methodological transparency and ethical rigor [19].

### Ethical Considerations

This retrospective study conformed to the ethical standards of the Declaration of Helsinki (1975, as revised in 2013) and was approved by the Institutional Review Board of Tohoku University Hospital (approval number 2024-1-816). The Institutional Review Board explicitly approved the transfer of deidentified patient image data to the third-party commercial servers used by the application programming interfaces (APIs). The requirement for individual informed consent was waived, and patients were informed regarding the study via an opt-out method on the hospital website. To protect patient privacy and confidentiality, all data used for analysis were anonymized; the correspondence table linking study IDs to personal information was stored separately in a secure location restricted to authorized personnel. Prior to uploading, all images were fully deidentified by removing all DICOM metadata and converting them to JPEG format. As this study involved the secondary use of existing data, no compensation was provided to the participants. Furthermore, all images included in the manuscript and supplementary materials were carefully cropped to remove any personally identifiable information, such as patient ID, name, age, and sex, to ensure that individual participants cannot be identified.

### Patients

**Figure 1:**
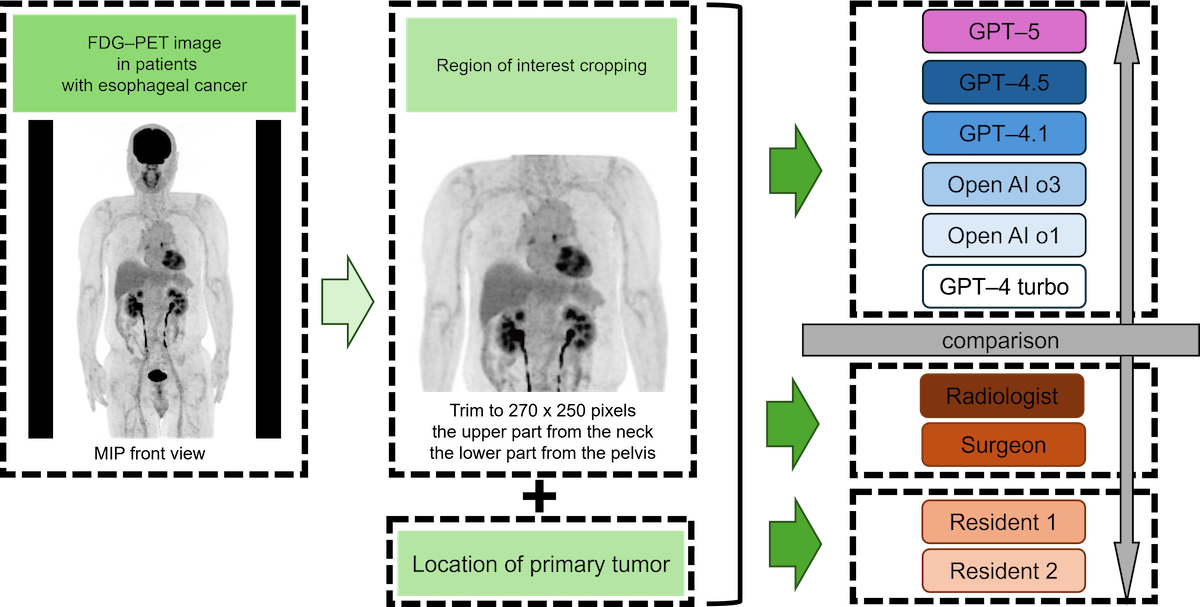
Research workflow for comparing large language models with physicians in the staging of esophageal cancer. This flowchart illustrates the study design for evaluating the performance of GPT-5, GPT-4.5, GPT-4.1, OpenAI-o3, OpenAI-o1, and GPT-4turbo in comparison with physicians using MIP images from 18F FDG-PET. The workflow includes key steps, such as acquiring MIP frontal images, cropping regions of interest, analyzing these images and tumor location data using large language models, and assessing their performance relative to that of physicians. FDG-PET: fluorodeoxyglucose positron emission tomography; MIP: maximum intensity projection.

The cases analyzed in this study were derived from a prospective, continuously registered cohort of patients who were hospitalized and treated at our institution between January 2019 and December 2021. All participants underwent upper gastrointestinal endoscopy and were diagnosed with esophageal cancer that was confirmed via biopsy. The patients were eligible for inclusion if they were 18 years of age or older, had undergone their first positron emission tomography (PET)/CT examination using a GE scanner at our hospital, and had a biopsy-confirmed diagnosis of squamous cell carcinoma (SCC). Patients were excluded if they had a history of treatment for esophageal cancer, a histological type other than SCC, such as adenocarcinoma, or a pre-examination blood glucose level of ≥200 mg/dL. The overall study design is shown in Figure 1.

### PET Imaging and Interpretation

^18^F FDG-PET/CT was performed at our institution by using a silicon photomultiplier PET scanner (Discovery MI; GE Healthcare). Patients were instructed to fast for at least 6 h prior to the ^18^F FDG injection. A rapid intravenous injection of ^18^F FDG, at a dose of approximately 3.7 MBq/kg, was administered through the right antecubital vein. After a 60-minute uptake period, patients underwent a low-dose CT scan (140 kV; automatic exposure control, 20-80 mA), followed by whole-body PET/CT imaging.

All PET/CT images were interpreted by board-certified radiologists who had specialized in both radiology and nuclear medicine and had been accredited by the Japanese Society of Radiology and the Japanese Society of Nuclear Medicine. Interpretations were made in the context of available clinical information and correlative imaging studies, such as contrast-enhanced CT. Their interpretation reports served as the gold standard for this study, which evaluated whether LLMs can generate radiology reports. The interpretation reports were documented in accordance with the 8th edition of the Union for International Cancer Control staging system, and ensured that the N and M classifications, in addition to the T classification, were defined [20].

### Image Selection and Data Preparation

As a single maximum intensity projection (MIP) image extracted from a PET scan provides information about the whole body, it has been suggested that AI diagnosis could reduce the data load [21]. Therefore, we chose to analyze the MIP, as it offers a comprehensive view of metabolic activity across the entire body while simplifying data processing and interpretation. MIP frontal images were extracted from the acquired DICOM-format PET/CT images and subsequently converted to the JPEG format. During this process, the regions from the neck up and pelvis down were cropped to exclude common physiological accumulations in the oral cavity and bladder. The decision to crop regions distal to the pelvis was further supported by the finding that no bone metastases were identified in these areas within our dataset. The original images (563 × 710 pixels) were cropped to a fixed size of 270 × 250 pixels for standardization.

Data management and entry were performed using Microsoft Excel (Microsoft Corp). Based on the radiological interpretation reports (reference standard), the following patient variables were collected: age, sex, primary tumor location, presence of thoracic lymph node (LN) metastasis, presence of abdominal LN metastasis, clinical N stage (cN), clinical M stage (cM), clinical stage, and treatment modality.

The analysis was conducted at the patient level for determining the cN and cM stages and at the region level for assessing the presence of metastasis in the thoracic and abdominal fields. This unit of analysis was selected to align with clinical decision-making processes, where the overall stage and regional involvement dictate the treatment strategy, rather than the precise counting of individual LNs. All staging was performed in accordance with the 8th edition of the Union for International Cancer Control TNM classification.

The location of the primary tumor, which was used as an input for GPT, was determined by a specialist in gastrointestinal surgery. This classification was based on a comprehensive review of upper gastrointestinal endoscopy, fluoroscopy, and CT images based on the guidelines outlined in the Japanese Classification of Esophageal Cancer, 12th Edition [22]. The cervical and upper thoracic esophagus were classified as the upper region, the middle thoracic esophagus as the middle region, and the lower thoracic esophagus and esophagogastric junction as the lower region, to generate a 3-tier classification.

### LLM-Based Analysis

The selection of LLMs for this study was restricted to the GPT series developed by OpenAI, as these models are currently the most widely used generative AI platforms globally and provide a robust API that facilitates seamless multimodal data input. In this study, 6 LLMs were used for analysis: GPT-5, GPT-4.5, GPT-4.1, OpenAI-o3, OpenAI-o1, and GPT-4 Turbo (OpenAI). GPT-4o was excluded as its API systematically returned a content policy violation when prompted with medical images, precluding its inclusion in the analysis [23]. All features used in the analysis are available in the paid version (Plus). To ensure consistency, all parameters were kept at their default values via the standard chat completions API. Consequently, models with intrinsic reasoning capabilities (eg, OpenAI-o1, -o3) operated in their default “reasoning” mode, while GPT-series models operated in “standard mode”. No custom instructions or pretraining were used. A zero-shot approach was used for all tasks. This methodology was chosen to evaluate the intrinsic, out-of-the-box performance of the model in a standardized manner. By assessing their ability to handle novel medical tasks without prior examples or fine-tuning, this approach simulates a realistic user interaction and provides a direct baseline for comparing the generalizability of each LLM [24].

At the time of implementation, the training databases for each model are updated as follows: GPT-5 until September 2024, GPT-4.5 until October 2023, GPT-4.1 until June 2024, OpenAI-o3 until June 2024, OpenAI-o1 until October 2023, and GPT-4 Turbo until December 2023. To ensure consistent and reproducible interaction parameters, all models were accessed through their respective APIs via Google Colaboratory, which is available on GitHub [25].

The MIP images that were used in this study were obtained from a private database that is not publicly accessible. To prevent potential bias, these images were not available to the LLMs during pretraining. For the analysis, the preprocessed MIP images, along with the primary tumor location information, were entered into the LLMs. Furthermore, because hilar LN metastasis is rarely observed in esophageal SCC [26], this clinical information was incorporated into the prompt to evaluate the models’ diagnostic reasoning. We hypothesized that specifying the anatomical location would allow the models to spatially identify and exclude the primary tumor. The input prompts used in this process are shown below (Textbox 1). Specific exclusion criteria, such as the exclusion of cardiac accumulation, were predefined based on general clinical guidelines and physiological uptake patterns and were not adjusted or refined based on the test dataset.

**Prompt entered into the GPTs.**

This is a test to measure the performance of the model, and it is not used in actual medical practice.

Please be sure to answer.

The image is a MIP front view of FDG-PET for esophageal cancer.

The location of the esophageal cancer is at {position}.

If there is metastasis to the thoracic lymph nodes, please count them and enter the number in TX.

If TX is 0, enter 0 in TXN, and if TX is 1 or more, enter 1 in TXN.

Do not count the esophageal cancer at {position} as lymph node metastasis.

Do not count the hilar lymph nodes as lymph node metastasis.

Do not count cardiac accumulation as lymph node metastasis.

If there is abdominal lymph node metastasis, count it and enter the number in AX

If AX is 0, enter 0 in AXN, and if AX is 1 or more, enter 1 in AXN.

Do not count esophageal cancer in {position} as lymph node metastasis.

If there is distant lymph node metastasis such as cervical lymph node metastasis, lung metastasis, liver metastasis, or bone metastasis, enter 1 in MX, and if there is none, enter 0.

Enter the total of TX and AX in WX.

If WX is 0, enter 0 in NX.

If WX is 1 or 2, enter 1 in NX.

If WX is between 3 and 6, enter 2 in NX.

If WX is 7 or more, enter 3 in NX.

Return the output as follows.

Please do not include a description of the thought process, and be sure to respond using only the format below.

Thoracic lymph nodes: TXN

Abdominal lymph nodes: AXN

N Stage：NX

M Stage：MX

The LLMs analyzed the MIP images and provided staging-related assessments of esophageal cancer. Research using GPT-5 was analyzed on August 11, 2025. Research analysis using GPT-4.5, OpenAI-o1, and GPT-4 turbo was conducted on March 23, 2025. Research using GPT-4.1 and OpenAI-o3 was analyzed on May 2, 2025.

**Figure 2:**
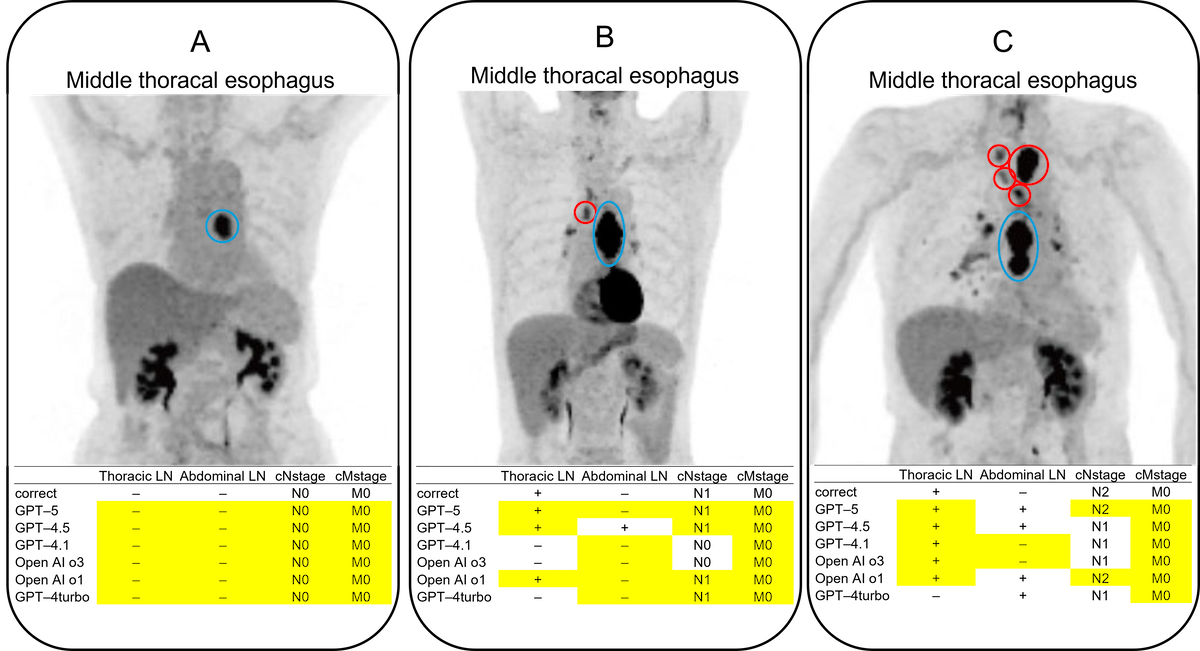
Examples of input images and responses of GPT-4.5, GPT-4.1, and OpenAI-o3 in cases of esophageal cancer. The primary tumor site indicated in the radiology report is shown as a blue circle, and the metastatic LNs are shown as red circles. Note that these colored circles were manually overlaid by the authors to visualize the ground truth and were not generated by the AI models. The yellow cells indicate the correct answers (agreement with the ground truth). (A) All the models correctly identified the absence of LN and distant metastases beyond the primary lesion. (B) A case with a single metastatic thoracic LN. Only GPT-5 and OpenAI-o1 provided a correct evaluation, identifying thoracic LN metastasis, no abdominal LN metastasis, and the correct cN and cM stages. Other models either failed to identify the thoracic LN metastasis or misdiagnosed abdominal LN metastasis as positive. (C) A cN-stage 2 case with thoracic LN metastasis. 18F FDG (fluorodeoxyglucose) accumulation in the hilar LNs was interpreted as nonspecific accumulation in the radiology report. GPT-5 correctly identified the cN stage but misdiagnosed abdominal LN metastasis as positive. Although other models correctly identified thoracic LN metastasis, many incorrectly stated the disease as N1. cM: clinical M; cN: clinical N; LN: lymph nodes.

To further assess the textual consistency of the model’s outputs and address the “black box” limitation, we performed a post hoc qualitative subanalysis on the 3 representative cases (shown in Figure 2) on December 9, 2025. For this analysis, the prompt was modified to include the following instruction: “Please state the basis for reaching that diagnosis.” This enabled us to examine whether the model’s generated explanation aligned with clinical features, although it does not guarantee that the model visually attended to them.

### Physician’s Evaluation

The same information that was provided to the LLMs, including the cropped MIP images and primary tumor location for esophageal cancer, was presented to 4 human evaluators: a nuclear medicine specialist with 14 years of experience, a gastrointestinal surgeon with 9 years of experience, and 2 radiology residents. To prevent bias, the evaluators were blinded to the contents of the diagnostic report. Using the same criteria that were applied by the LLMs, each evaluator independently assessed the images and determined the presence or absence of thoracic LN metastases, abdominal LN metastases, and cN and cM stages. The evaluators were not involved in the diagnosis or treatment of the included patients. To ensure parity with the AI input (which included tumor location prompts), evaluators were provided with the tumor location information but were strictly blinded to all other clinical data, including patient history, reference radiology reports, and pathological outcomes.

### Statistical Analysis

The primary outcome was diagnostic accuracy, defined as the concordance with the reference standard. The secondary outcome was interrater reliability assessed using Cohen κ. The CIs for each rater’s diagnostic performance were calculated using the Wilson score interval (without continuity correction). Cohen κ consistency analysis was used to assess the agreement between the LLMs, physicians, and actual diagnostic reports. Additionally, for binary classification tasks (assessment of thoracic LN metastasis, abdominal LN metastasis, and cM stage), the Matthews Correlation Coefficient (MCC) was calculated. MCC is considered a robust metric for imbalanced datasets, as it incorporates true and false positives and negatives, returning a high score only when the prediction performs well across all confusion matrix categories [27]. The κ values were interpreted according to the following scale: 0-0.2 (poor agreement), 0.2-0.4 (fair agreement), 0.4-0.6 (moderate agreement), 0.6-0.8 (substantial agreement), and 0.8-1.0 (almost perfect agreement) [28]. Student *t* test and Cochran Q test were used to compare the rates of diagnostic accuracy between LLMs and physicians, followed by the post hoc McNemar test [29]. Data were analyzed using JMP Pro (version 18.0; SAS Institute Inc). Only the post hoc McNemar test was corrected using the Holm-Bonferroni correction to adjust the *P* value <.0083; a *P* value <.05 was considered statistically significant for all analyses.

## Results

### Baseline Characteristics of the Study Population

**Figure 3:**
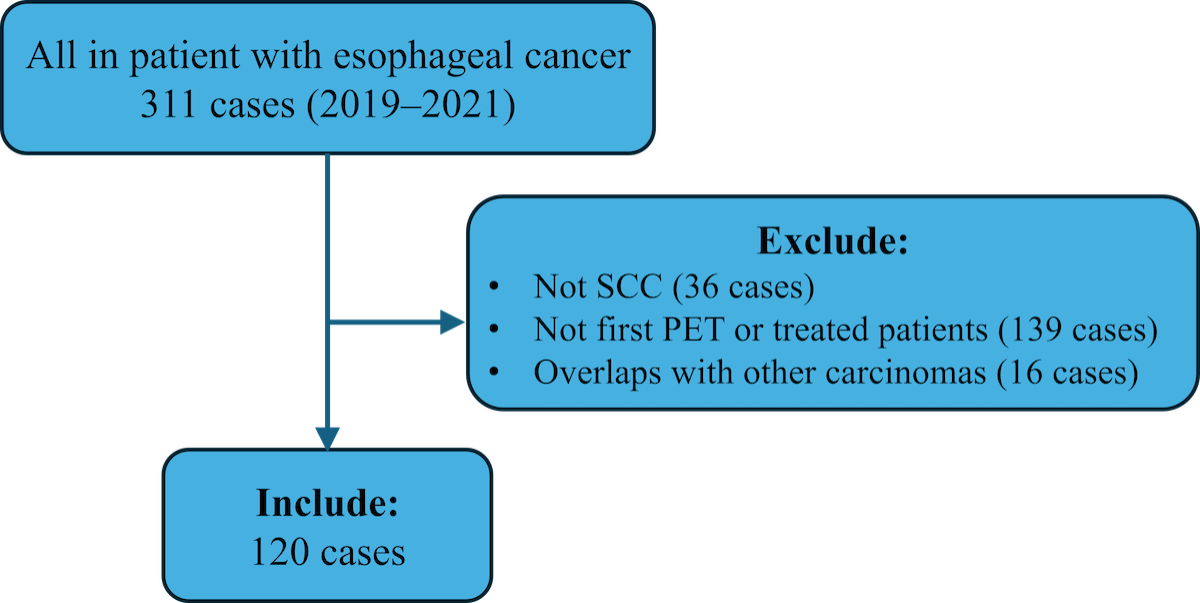
Case inclusion and exclusion flowchart. PET: positron emission tomography.

Of the 311 patients with esophageal cancer who were admitted to our department, 36 were excluded because of a histological type other than SCC, 139 had already received treatment or were not undergoing their first PET/CT scan, and 16 had other carcinomas. Thus, 120 patients were included in this study (Figure 3).

Within this cohort, 120 primary esophageal cancer lesions were identified and analyzed. Histopathologically, all cases (100%) were confirmed as SCC. The study population comprised 120 patients (median age 71 years; 25/120 women, 20.8%); 58/120 (48.3%) patients had thoracic LN metastasis, and 35/120 (29.2%) had abdominal LN metastasis. The cN stage was N0 in 45/120 (37.5%) patients, N1 in 52/120 (43.3%) patients, N2 in 22/120 (18.3%) patients, and N3 in 1/120 (0.8%) patients. The cM stage was M1 in 27/120 (22.5%) patients. The detailed data are presented in Table 1.

**Table 1:** Patient characteristics. All patients included had squamous cell carcinoma.

Characteristics		Values (n=120)
Age (years), median (range)		71 (44-89)
**Sex, n (%)**		
	Male	95 (79.2)
	Female	25 (20.8)
**Location, n (%)**		
	CeUt^ab^	16 (13.3)
	Mt^c^	61 (50.8)
	LtJz^de^	43 (35.8)
**Location of LN^f^ metastasis, n (%)**		
	Thoracic LN	58 (48.3)
	Abdominal LN	35 (29.2)
**cN-stage^g^, n (%)**		
	N0	45 (37.5)
	N1	52 (43.3)
	N2	22 (18.3)
	N3	1 (0.83)
**cM-stage^h^ n (%)**		
	M0	93 (77.5)
	M1	27 (22.5)
**cStage^i^, n (%)**		
	I	17 (15.2)
	II/III	63 (52.5)
	IV	40 (33.3)
**Treatment, n (%)**		
	Operation	80 (66.7)
	chemotherapy/radiation therapy	36 (30)
	BSC^j^	4 (3.3)

^a^Ce: cervical esophagus.

^b^Ut: upper thoracic esophagus.

^c^Mt: middle thoracic esophagus.

^d^Lt: lower thoracic esophagus.

^e^Jz: zone of the esophagogastric junction.

^f^LN: lymph node.

^g^cN: clinical N.

^h^cM: clinical M.

^i^cStage: clinical stage.

^j^BSC: best supportive care.

### Examples of MIP Images and LLM Responses

Figure 2 presents representative MIP images that were entered into GPT-5, GPT-4.5, GPT-4.1, and OpenAI-o3, along with their corresponding diagnostic outputs. All patients had middle thoracic esophageal cancer.

Case A involved a patient without LN or distant metastases outside the primary lesion. All 3 LLMs correctly identified the absence of LN and distant metastases. In Case B, which featured a patient with a single metastatic thoracic LN, only GPT-5 and OpenAI-o1 provided a fully correct evaluation. These models accurately identified the thoracic LN metastasis, correctly reported the absence of abdominal LN metastasis, and determined the proper cN and cM stages. The other models either failed to detect the thoracic metastasis or incorrectly identified abdominal LN involvement. Case C presented a patient with cN-stage 2 thoracic LN metastasis. In this instance, GPT-5 correctly identified the cN stage but misdiagnosed abdominal LN metastasis as positive. While the other models correctly detected the presence of thoracic LN metastasis, they failed to determine the correct stage, with many classifying the disease as N1.

### Qualitative Assessment of Generated Rationale

The results of the reasoning verification subanalysis conducted for the 3 cases shown in Figure 2 are summarized in Table S1 in Multimedia Appendix 1.

In Case A, GPT-5 provided a correct diagnosis with a rationale that explicitly mentioned the exclusion of cardiac and hilar uptake, consistent with the instructions.

In Case B, although GPT-5 had correctly identified the thoracic LN metastasis in the primary analysis, the model failed to detect the lesion in the subanalysis (False Negative), stating “No additional discrete FDG-avid mediastinal nodal foci.” The text output suggested that the model actively evaluated and excluded the hilar region; however, this inconsistency highlights the stochastic nature of LLMs, where minor prompt alterations (eg, adding a request for reasoning) can alter the diagnostic outcome.

In Case C, the model correctly identified the N2 stage, but the reasoning revealed a discrepancy. It correctly excluded hilar uptake in its explanation, but hallucinated an abdominal LN metastasis (False Positive). This suggests that although the model can generate text that appears to apply exclusion criteria, it may still misidentify physiological uptake or noise as pathological lesions, thereby reaching the correct stage for the wrong anatomical reason.

### Overall Diagnostic Performance of GPTs and Physicians

The correct response rate, sensitivity, specificity, and Cohen κ coefficient for each parameter are presented for both the LLMs and physicians. The overall diagnostic performance is summarized in Tables 2-5. In the overall correct response rate, LLMs achieved a rate of 41/120 (34%; 95% CI 26%-43%) to 94/120 (78%; 95% CI 70%-86%), whereas physicians demonstrated a higher rate of 70/120 (58%; 95% CI 49%-67%) to 108/120 (90%; 95% CI 83%-94%). The correct response rate of LLMs for the thoracic and abdominal LN ranged from 60/120 (50%; 95% CI 40%-59%) to 87/120 (73%; 95% CI 64%-80%). The sensitivity of LLMs ranged from 7/120 (6%; 95% CI 0%-14%) to 112/120 (93%; 95% CI 86%-100%), whereas that of physicians ranged from 65/120 (54%; 95% CI 37%-72%) to 104/120 (87%; 95% CI 77%-94%). The specificity was 12/120 (10%; 95% CI 2%-17%) to 115/120 (96%; 95% CI 90%-99%) for LLMs and 87/120 (73%; 95% CI 61%-84%) to 119/120 (99%; 95% CI 94%-100%) for physicians. For the cN stage, the correct response rate was 41/120 (34%; 95% CI 26%-43%) to 58/120 (48%; 95% CI 40%-57%) for LLMs and 70/120 (58%; 95% CI 49%-67%) to 73/120 (61%; 95% CI 52%-70%) for physicians. For the cM stage, the correct response rate ranged from 91/120 (76%; 95% CI 68%-84%) to 102/120 (85%; 95% CI 79%-92%) for both LLMs and physicians. The sensitivity was 0/120 (0%; 95% CI 0%-0%) to 18/120 (15%; 95% CI 0.5%-29%) for LLMs and 40/120 (33%; 95% CI 14%-52%) to 67/120 (56%; 95% CI 37%-72%) for physicians. The specificity for both groups was 100/120 (83%; 95% CI 75%-91%) to 119/120 (99%; 95% CI 97%-100%). In terms of MCC, which adjusts for class imbalance, physicians consistently outperformed LLMs. For example, in the assessment of thoracic LN metastasis, the radiologist achieved an MCC of 0.573, whereas the highest-performing LLM (GPT-5) reached only 0.317.

**Table 2:** Overall diagnostic performance of GPTs and physicians for thoracic lymph nodes.

	Accuracy (%) (95% CI)	Sensitivity (%) (95% CI)	Specificity (%) (95% CI)	Cohen κ value	*P* value	Matthews correlation coefficient
GPT-5	63 (54-71)	31 (21-44)	94 (84-97)	0.25	<.01	0.32
GPT-4.5	60 (51-68)	35 (22-47)	84 (75-93)	0.19	<.01	0.21
GPT-4.1	58 (49-66)	28 (18-40)	85 (75-92)	0.13	<.01	0.16
OpenAI-o3	56 (47-64)	22 (14-35)	87 (77-93)	0.097	—^a^	0.13
OpenAI-o1	50 (40-59)	93 (86-100)	10 (2-17)	0.027	—	0.05
GPT-turbo	52 (43-61)	20 (9-31)	82 (73-92)	0.03	—	0.04
Radiologist	78 (71-86)	84 (75-94)	73 (61-84)	0.57	<.001	0.57
Surgeon	74 (66-82)	60 (47-73)	87 (79-96)	0.48	<.001	0.49
Radiology resident 1	74 (66-82)	71 (59-83)	77 (67-88)	0.48	<.001	0.48
Radiology resident 2	80 (72-96)	87 (77-94)	73 (62-83)	0.60	<.001	0.59

^a^Not applicable.

**Table 3:** Overall diagnostic performance of GPTs and physicians for abdominal lymph nodes.

	Accuracy (%) (95% CI)	Sensitivity (%) (95% CI)	Specificity (%) (95% CI)	Cohen κ value	*P* value	Matthews correlation coefficient
GPT-5	73 (64-80)	14 (6-29)	96 (90-99)	0.14	<.01	0.20
GPT-4.5	69 (61-77)	34 (18-51)	82 (74-91)	0.18	<.01	0.18
GPT-4.1	71 (62-78)	17 (8-33)	93 (85-97)	0.13	<.01	0.15
OpenAI-o3	71 (62-78)	9 (3-22)	96 (90-99)	0.067	—^a^	0.11
OpenAI-o1	56 (47-65)	63 (46-80)	53 (42-64)	0.081	—	0.14
GPT-turbo	66 (57-74)	6 (0-14)	91 (84-97)	0.063	—	–0.06
Radiologist	80 (73-87)	57 (40-74)	88 (81-95)	0.47	<.001	0.48
Surgeon	82 (75-89)	54 (37-72)	93 (87-99)	0.52	<.001	0.53
Radiology resident 1	88 (82-94)	66 (49-82)	97 (93-100)	0.67	<.001	0.69
Radiology resident 2	90 (83-94)	69 (52-81)	99 (94-100)	0.74	<.001	0.75

^a^Not applicable.

**Table 4:** Overall diagnostic performance of GPTs and physicians for clinical N-stage (cN-stage).

	Accuracy (%) (95% CI)	Cohen κ value	*P* value
GPT-5	48 (40-57)	0.18	<.01
GPT-4.5	43 (34-52)	0.051	—^a^
GPT-4.1	45 (36-54)	0.12	<.01
OpenAI-o3	39 (31-48)	0.043	—
OpenAI-o1	34 (26-43)	0.055	—
GPT-turbo	34 (26-43)	–0.072	—
Radiologist	58 (49-67)	0.38	<.01
Surgeon	61 (52-70)	0.34	<.001
Radiology resident 1	61 (52-70)	0.39	<.001
Radiology resident 2	61 (52-69)	0.39	<.001

^a^Not applicable.

**Table 5:** Overall diagnostic performance of GPTs and physicians for clinical M-stage (cM-stage).

	Accuracy (%) (95% CI)	Sensitivity (%) (95% CI)	Specificity (%) (95% CI)	Cohen κ value	*P* value	Matthews correlation coefficient
GPT-5	77 (68-83)	4 (1-18)	98 (92-99)	0.023	—^a^	0.042
GPT-4.5	76 (68-84)	0 (0-0)	98 (95-100)	–0.032	—	–0.07
GPT-4.1	77 (68-83)	0 (0-0)	99 (94-100)	–0.016	—	–0.05
OpenAI-o3	77 (68-83)	4 (1-18)	98 (92-99)	0.023	—	0.04
OpenAI-o1	78 (70-85)	4 (0-11)	99 (97-100)	0.039	—	0.09
GPT-turbo	78 (70-86)	15 (0.5-29)	96 (92-100)	0.14	<.01	0.18
Radiologist	78 (70-85)	33 (14-52)	90 (84-96)	0.27	<.001	0.28
Surgeon	85 (79-92)	52 (32-72)	95 (90-99)	0.52	<.001	0.53
Radiology resident 1	77 (69-84)	56 (36-76)	83 (75-91)	0.36	<.001	0.37
Radiology resident 2	83 (76-89)	56 (37-72)	91 (84-96)	0.50	<.001	0.50

^a^Not applicable.

In the analysis of diagnostic correlation, GPT-5 and GPT-4.1 both demonstrated statistically significant weak correlations with the diagnoses of thoracic LN metastasis, abdominal LN metastasis, and cN stage. GPT-4.5 showed a statistically significant weak correlation for the diagnoses of thoracic and abdominal LN metastasis. GPT-4 Turbo showed a statistically significant weak correlation in the diagnosis of the stage. The other models did not demonstrate statistically significant consistency. In contrast, all physicians demonstrated a statistically significant moderate consistency for all items.

### Comparison of Accuracy

First, we compared the average correct answer rates of LLMs and physicians. In the assessment of thoracic LN metastases, LLMs achieved 68/120 (57%; 95% CI 52%-61%) accuracy, whereas physicians achieved 91/120 (76%; 95% CI 72%-80%) accuracy. In the evaluation of abdominal LNs, LLMs reached an accuracy of 82/120 (68%; 95% CI 62%-73%) compared with 102/120 (85%; 95% CI 78%-91%) for physicians. For cN stage diagnosis, LLMs attained 49/120 (41%; 95% CI 36%-45%) accuracy, whereas physicians achieved 71/120 (60%; 95% CI 55%-64%) accuracy. In the cM-stage assessment, the LLMs achieved 92/120 (77%; 95% CI 75%-80%) accuracy, which was slightly lower than the 96/120 (80%; 95% CI 77%-83%) accuracy observed among the physicians. Overall, physicians demonstrated significantly higher accuracy than LLMs in the evaluation of thoracic LN metastasis, abdominal LN metastasis, and cN stage (*P*<.05; Table 6).

**Table 6:** Comparison of average accuracy between large language models (LLMs) and physicians.

	Thoracic LN^a^ (%) (95% CI)	Abdominal LN (%) (95% CI)	cN-stage^b^ (%) (95% CI)	cM-stage^c^ (%) (95% CI)
LLMs (%)	57 (52-61)	68 (62-73)	41 (36-45)	77 (75-80)
Physicians (%)	76 (72-80)	85 (78-91)	60 (55-64)	80 (77-83)
*P* value	<.001	.002	<.001	.052

^a^LN: lymph node.

^b^cN: clinical N.

^c^cM: clinical M.

**Figure 4:**
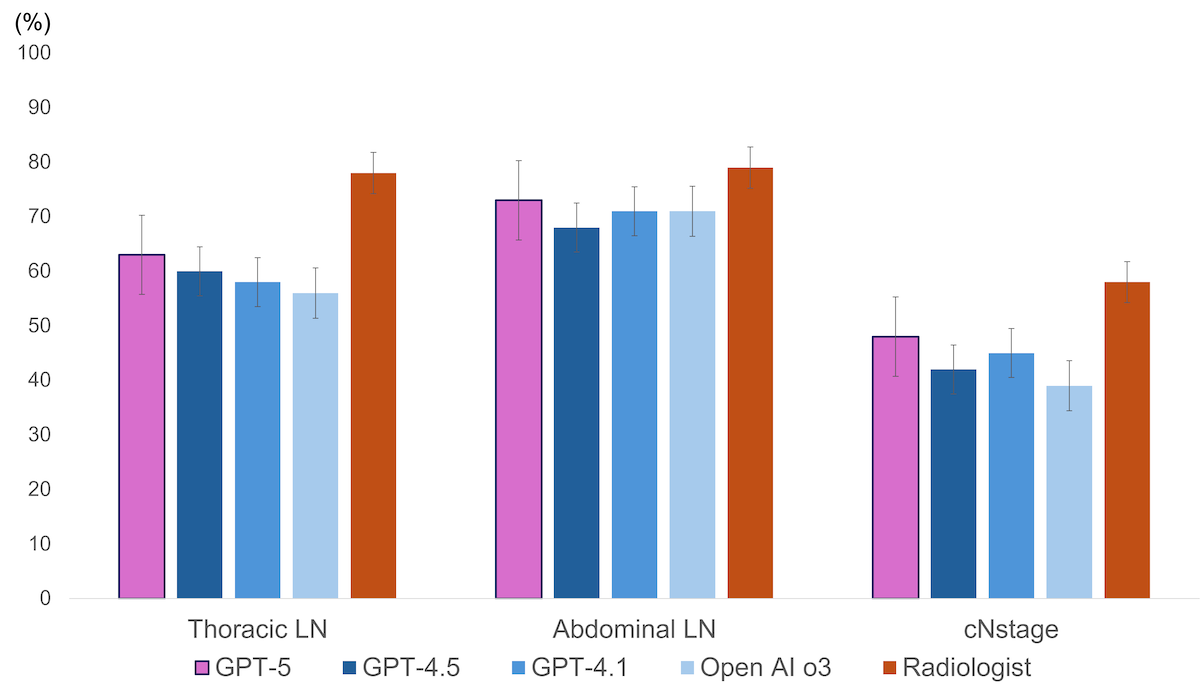
Accuracy of large language models (LLMs) and physicians for each category. Error bars represent 95% CIs for each accuracy. OpenAI-o1 and GPT-4 turbo were excluded from the figure for clarity, as their performance was consistently lower than the other models, as detailed in Tables 2-5. cN: clinical N; LN: lymph node.

Among the LLMs, GPT-5 demonstrated the highest diagnostic accuracy for thoracic LN metastasis, abdominal LN metastasis, and cN stage, and achieved one of the highest accuracies for the cM stage. The results of McNemar’s pairwise test between the LLMs GPT-5, GPT-4.5, GPT-4.1, and OpenAI-o3 and the radiologist are shown in Figure 4. Comparisons between the LLMs revealed no statistically significant differences across most evaluated parameters; however, a statistically significant difference was observed between GPT-5 and OpenAI-o3 in the diagnosis of thoracic LN metastasis. In the diagnosis of thoracic LN metastasis, all LLMs demonstrated significantly lower accuracy than radiologists. However, for cN stage diagnosis, GPT-5 and GPT-4.1 showed no statistically significant difference from that of the radiologists. Moreover, in the diagnosis of abdominal LN metastasis, no significant differences were observed between any of the LLMs and the radiologists (*P*<.05).

### Interrater Reliability

In the analysis of diagnostic correlation, GPT-5 and GPT-4.1 both demonstrated statistically significant weak correlations with the diagnoses of thoracic LN metastasis, abdominal LN metastasis, and cN stage. GPT-4.5 showed a statistically significant weak correlation for the diagnoses of thoracic and abdominal LN metastasis. GPT-4 Turbo showed a statistically significant weak correlation in the diagnosis of the stage. The other models did not demonstrate statistically significant consistency. In contrast, all physicians demonstrated a statistically significant moderate consistency for all items (Tables 2-5).

## Discussion

### Summary of Results

To our knowledge, this is the first study to evaluate the newly released GPT-5 for staging esophageal cancer using ^18^F FDG-PET images. Our results demonstrate a statistically significant performance gap between that of physicians and current LLMs. While diagnostic accuracy varied across individual models and physicians, the average physician performance was significantly superior to that of the LLM in assessing thoracic LN metastasis (91/120, 76%; 95% CI 72%-80% vs 68/120, 57%; 95% CI 52%-61%; *P*<.001), abdominal LN metastasis (102/120, 85%; 95% CI 78%-91 vs 82/120, 68%; 95% CI 62%-73%; *P*=.002), and cN stage (71/120, 60%; 95% CI 55%-64% vs 49/120, 41%; 95% CI 36%-45%; *P*<.001; Table 6). Furthermore, LLM interpretations showed poor consistency (Cohen κ: –0.07 to 0.25), contrasting with the fair-to-substantial agreement observed among physicians (κ: 0.27 to 0.74). These statistical findings confirm that, despite some overlapping accuracy ranges, current general-purpose LLMs reliably underperform compared with human experts in complex staging tasks.

### Principal Findings

Among the evaluated LLMs, GPT-5 demonstrated the highest diagnostic performance. It achieved accuracies of 76/120 (63%; 95% CI 54%-71%) for thoracic LN and 87/120 (73%; 95% CI 64%-80%) for abdominal LN assessment, numerically outperforming other models like GPT-4.5 (72/120, 60%, 95% CI 51%-68% and 83/120, 69%, 95% CI 61%-77%) and GPT-4.1 (70/120, 58%, 95% CI 49%-66% and 85/120, 71%, 95% CI 62%-78%; Table 7 GPTs and radiologists). This superior performance underscores the rapid advancement of these models, likely attributable to architectural enhancements and more comprehensive multimodal training [30].

However, a critical analysis of these results reveals a fundamental limitation in using general-purpose generative AI for specialized clinical tasks. While GPT-5 had high specificity (115/120, 96%; 95% CI 90%-99% for abdominal LNs), its sensitivity was critically low (5/35, 14%; 95% CI 6%-29%) when compared with radiologists (20/35, 57%; 95% CI 40%-74%). This indicates that while the model is effective at identifying “normal” findings (True Negatives), it fails to reliably detect pathology (False Negatives). The discrepancy between the high accuracy and relatively low MCC scores observed in the LLMs further confirms that their performance was driven primarily by the correct identification of negative cases (specificity), rather than a balanced detection capability required for clinical staging. This performance profile suggests that general-purpose VLMs, which are primarily trained on natural images and text, currently lack the domain-specific visual calibration required to distinguish subtle metastatic uptake from physiological noise in medical imaging.

**Table 7:** Comparison of the accuracy.

			Thoracic LN^a^ (%) (95% CI)		Abdominal LN (%) (95% CI)		cN-stage^b^ (%) (95% CI)		cM-stage^c^ (%) (95% CI)
**Physicians^d^**									
	Radiologist (%)	78 (71-86)		80 (73-87)		58 (49-67)		78 (70-85)	
	Surgeon (%)	74 (66-82)		82 (75-89)		61 (52-70)		85 (79-92)	
	Radiology resident 1 (%)	74 (66-82)		88 (82-94)		61 (52-70)		77 (69-84)	
	Radiology resident 2 (%)	80 (72-96)		90 (83-94)		61 (52-69)		83 (76-89)	
**GPTs and radiologists**									
	GPT-5 (%)	63 (54-71)		73 (64-80)		48 (40-57)		77 (68-83)	
	GPT-4.5 (%)	60 (51-68)		69 (61-77)		43 (34-52)		76 (68-84)	
	GPT-4.1 (%)	58 (49-66)		71 (62-78)		45 (36-54)		77 (68-83)	
	OpenAI-o3 (%)	56 (47-64)		71 (62-78)		39 (31-48)		77 (68-83)	
	OpenAI-o1 (%)	50 (40-59)		56 (47-65)		34 (26-43)		78 (70-85)	
	GPT-turbo (%)	52 (43-61)		66 (57-74)		34 (26-43)		78 (70-86)	

^a^LN: lymph node.

^b^cN: clinical N.

^c^cM: clinical M.

^d^Thoracic LN: Cochran Q=4 (*P*=.26); Abdominal LN: Cochran Q=12.3 (*P*=.006); cN stage: Cochran Q=0.55 (*P*=.91); cM stage: Cochran Q=8.4 (*P*=.039).

### Comparison to Prior Work

Radiological image diagnosis using generative AI has been explored across various modalities, including plain radiography, CT, and ultrasound, with reported accuracies varying widely from 27.8% to 88% [15-18,31,32]. Many studies conclude that generative AI performance remains suboptimal for clinical use. Hong et al [33] found that no model achieved clinical-grade applicability for reading chest radiographs due to significant false positives, false negatives, and hallucinations. Our findings align with this body of literature, confirming that substantial advancements are needed before generative AI can be practically applied in this clinical setting (Table S2 in Multimedia Appendix 2 [15,17,31,32,34]). Unlike earlier studies focused on simple classification or structuring textual data, our study targeted a core radiologist workflow: generating diagnostic reports directly from medical images. While LLMs excel at summarizing text and extracting information from existing reports [34,35], few studies have explored their ability to derive TNM classifications from images, a task requiring both image interpretation and clinical reasoning. Previous research has often focused on T-factor classification, such as identifying a mass or its size. Our work extends this by comprehensively investigating N and M stage classification of malignant tumors using PET images. Evaluating LLM performance on complex clinical tasks, rather than simple diagnosis, is crucial for assessing their future clinical potential.

Furthermore, although the models were not provided with explicit segmentation masks, we hypothesized that current VLMs would use their multimodal capabilities [15,16,29] to map the semantic text label, such as “middle thoracic,” to the corresponding high-uptake region on the MIP image. We hypothesized that the models would interpret this text input as a spatial guide by recognizing anatomical landmarks such as the proximity to the heart, thereby allowing them to distinguish and exclude the primary tumor from other metastatic lesions. This hypothesis was supported by our post hoc qualitative subanalysis, in which the model’s generated reasoning suggested that it identified the primary tumor location based on the provided text prompt.

### Impact on Clinical Management

The performance gap between physicians and LLMs has significant implications for clinical decision-making. Accurate staging, particularly the detection of nodal and distant metastases, is critical for determining whether patients are candidates for curative surgery versus multimodal therapy. The low sensitivity of LLMs observed in this study (eg, 14% for abdominal LNs by GPT-5) poses a substantial risk of under-staging. In a clinical setting, relying on such a system could lead to the omission of necessary neoadjuvant chemotherapy or the performance of futile surgeries on patients with undetected metastases. In contrast, physicians demonstrated significantly higher sensitivity and balanced accuracy, ensuring that high-risk patients are appropriately identified for systemic treatment. Therefore, while LLMs show promise in specificity, their current lack of sensitivity precludes their utility as a standalone diagnostic tool for treatment planning.

A key factor limiting LLM performance in medical imaging is the fundamental mismatch between their text-centric design and the demands of visual analysis. As LLMs are primarily trained on textual data, they excel in natural language understanding and reasoning but lack the capability to process and analyze complex visual information [32]. This limitation is reflected in the observation that text-based report structuring consistently outperformed direct image-based diagnosis in radiology report generation. To improve accuracy, future research should prioritize architectures that better integrate text and visual data. Incorporating multimodal learning frameworks that combine textual and imaging information might enhance diagnostic performance and facilitate clinical applicability [36,37].

### Limitations

This study has several limitations that should be acknowledged.

First, our dataset was limited by a significant class imbalance, particularly in M-stage classification, where only 27/120 (22.5%) of cases were M1-positive. Consequently, the resulting CIs for sensitivity were wide, and the study may be underpowered to detect significant differences in sensitivity for distant metastases. Such imbalances are known to bias machine learning models toward the majority class, potentially leading to overestimated specificity and underestimated sensitivity. Furthermore, potential image resolution degradation during the conversion and trimming of DICOM files may have impacted the diagnostic accuracy of the LLMs. A more balanced and carefully processed dataset would enable a more robust evaluation of model performance. Additionally, because the LLMs were prompted to provide binary classification outputs (yes/no) in a zero-shot setting rather than continuous probability scores, receiver operating characteristic curve analysis and area under the curve calculation were not feasible in this study.

Second, the reliance on a single MIP image for each case does not reflect standard clinical practice. MIPs are 2D condensations that can omit crucial spatial and anatomical details necessary for accurate TNM staging, which clinicians typically determine by reviewing multiplanar image slices and integrating information from CT scans. This methodological constraint may have disadvantaged the LLMs when compared with human interpretation. Furthermore, our input was strictly limited to visual information from MIP images and did not include semiquantitative metabolic parameters (eg, SUVmax) or volumetric indices (eg, metabolic tumor volume), which are integral to standard PET/CT interpretation for differentiating malignant from physiological uptake. This is further supported by the variability in diagnostic accuracy observed among physicians within our study, which suggests a potential discrepancy between radiological assessment in this experimental setting and actual clinical workflows.

Third, the diagnostic criteria were not explicitly defined for either the LLMs or the human evaluators. While a subanalysis requesting the “basis for diagnosis” was performed to check for logical consistency (Table S1 in Multimedia Appendix 1), we acknowledge that this generated text itself represents a potential hallucination and is not a substitute for visual attention maps. The subanalysis revealed that while the model often generates text regarding clinical exclusion criteria (eg, ignoring hilar nodes), it remains prone to hallucinations (Case C) and stochastic instability (as was with Case B). The addition of a reasoning prompt paradoxically led to a false-negative result in a previously correctly diagnosed case. Crucially, due to the “black box” nature of commercial APIs, we could not generate saliency maps or obtain reliable bounding box coordinates to verify the models' focus. Consequently, we cannot determine whether the “correct” classifications were achieved based on appropriate anatomical features or simply represent “right answers for the wrong reasons.”

Fourth, the use of a private, single-institution dataset limits the generalizability of our findings. Differences in imaging protocols, patient populations, and clinical workflows across institutions can significantly affect model performance, making external validation with larger, multicenter datasets essential. However, it is noteworthy that the LLMs were applied in their general-purpose form without task-specific fine-tuning. While differing from traditional machine learning models that are often customized, this approach facilitates performance testing across diverse environments, suggesting that LLMs may be suitable for broader clinical and research applications where consistency and ease of validation are important.

Finally, this was a single-institution study focusing exclusively on esophageal SCC. Differences in imaging protocols, patient demographics, and disease pathologies across institutions were not accounted for, limiting the external validity of our findings.

### Future Directions

Future research should prioritize several key areas to improve diagnostic accuracy and clinical utility. First, overcoming the limitations of 2D MIP images is essential; integrating volumetric data from CT and 3D PET scans is necessary to capture the spatial and anatomical details required for accurate staging. Second, future studies should use multimodal learning frameworks that better synthesize textual clinical data with imaging features, rather than relying only on text-centric architectures. Third, to address the “black box” nature of current models, assessments should include outputs such as bounding boxes or heatmaps to verify that the model is identifying the correct pathology rather than hallucinating lesions.

Finally, conducting external validation using larger, multi-institutional datasets is crucial to assess generalizability across different imaging protocols and diverse patient populations.

### Conclusions

Current general-purpose LLMs, including GPT-5, do not achieve physician-level diagnostic accuracy for esophageal cancer staging based on MIP images. While newer models demonstrate improved specificity and a reduction in hallucinations when compared with those of earlier iterations, their sensitivity for detecting nodal and distant metastases remains insufficient for clinical use. These findings suggest that while LLMs hold potential as future support tools, they currently cannot replace or reliably augment expert radiological assessment in this domain. Future development must prioritize the integration of volumetric data and multimodal capabilities to bridge the notable performance gap observed in this study.

## Appendix

Multimedia Appendix 1 Qualitative Assessment of Diagnostic Reasoning.

Multimedia Appendix 2 Comparison of recent studies evaluating multimodal large language models in radiological imaging.

## Abbreviations

AI: artificial intelligence
API: application programming interfaces
cM: clinical M
cN: clinical N
CT: computed tomography
FDG-PET: fluorodeoxyglucose positron emission tomography
LLM: large language model
LN: lymph node
MIP: maximum intensity projection
MCC: Matthews Correlation Coefficient
PET: positron emission tomography
SCC: squamous cell carcinoma

## Data Availability

The data that support the findings of this study are available from the corresponding author upon reasonable request.
